# The impact of gestational weeks of Coronavirus disease 2019 (COVID-19) infection on perinatal outcomes

**DOI:** 10.1186/s12978-024-01762-9

**Published:** 2024-03-04

**Authors:** Jiao Yi, Lei Chen, Xianglian Meng, Yi Chen

**Affiliations:** Department of Obstetrics and Gynecology, Maternal and Child Health Care Hospital Affiliated With Anhui Medical University, Anhui Maternal and Child Health Care Hospital, No 15 Yimin Street, Hefei, 230000 China

**Keywords:** COVID-19, SARS-CoV-2, Preeclampsia, Gestational diabetes mellitus, Gestational week of infection

## Abstract

**Background:**

To evaluate the relationship between coronavirus disease 2019 (COVID-19) infection at different time points during pregnancy and perinatal outcomes.

**Methods:**

This retrospective study included 611 women who hospitalized for delivery between December 7 and April 30, 2023. Based on the different pregnancy weeks infected with COVID-19, the participants were divided into four groups: Group 1 (14–27^+6^ weeks gestation), Group 2 (28–36^+6^ weeks gestation), Group 3 (37–39^+6^ weeks gestation), and Group 4 (≥ 40 weeks gestation). Data including maternal demographic characteristics, clinical profiles, and perinatal outcomes were analyzed.

**Results:**

There were no significant differences in maternal demographic characteristics among the four groups (*P* > 0.05). Compared to Groups 3 and 4, a higher rate of fever was noted in Groups 1 and 2 (*P* < 0.05). The frequency of preeclampsia and gestational diabetes mellitus showed a decreasing trend as pregnancy progressing (*P* < 0.05). Preterm delivery and neonatal intensive care unit admission were more frequently observed in Groups 1 and 2 than in Groups 3 and 4 (*P* < 0.05). Multivariate logistic regression analysis demonstrated that the timing of gestation in which COVID-19 was infected was not associated with preterm delivery and neonatal intensive care unit admission (*P* > 0.05), whereas gestational age at COVID-19 infection was negatively associated with the occurrence of preeclampsia and gestational diabetes mellitus (*P* < 0.05).

**Conclusions:**

Gestational age at COVID-19 infection is a simple parameter that predicts adverse perinatal outcomes to aid clinicians in determining to provide early enhanced prenatal care and increased monitoring to reduce maternal complications.

## Introduction

Over the past 3 years, the worldwide healthcare, social, and economical systems have been overwhelmed by the outbreak of coronavirus disease 2019 (COVID-19) pandemic [1]. The world has been fighting against the COVID-19 pandemic [2]. Severe acute respiratory syndrome-coronavirus-2 (SARS-CoV-2), the causative agent of COVID-19, has infected more than 500 million people, with more than 5.5 million estimated deaths. The number of new confirmed cases is growing daily, as the epidemic continue to spread [3]. Thus, COVID-19 has become a worldwide concern and unprecedented challenge for the healthcare systems. Researchers worldwide have been working on finding strategies for COVID-19 control and treatment [4], currently, no specific and effective treatment modality has been approved.

Theoretically, pregnant women are particularly susceptible to acquiring respiratory pathogens owing to adaptive physiological alterations in the immune, cardiovascular and respiratory systems [5], which in turn may increase the clinical severity of pneumonia in pregnant women [6]. Accumulating evidence has indicated that pregnant women infected with SARS-CoV-2 usually presents as asymptomatic or mildly symptomatic [7, 8], and the clinical course of SARS-CoV-2 infected pregnant women is similar to that of the general population [9] with low morbidity and mortality rates [10]. In contrast, conflicting results reporting that infection of COVID-19 during pregnancy may result in a higher risk of obstetric complications and adverse perinatal outcomes [11, 12], with significantly higher rates of pregnancy complications were observed in the severe-critical COVID-19 pregnant women [13]. Furthermore, most of the published data related to COVID-19 and pregnancy come from the analysis of small electronic health records and generally include only third-trimester pregnancies. To date, there is an absence of comparison between COVID-19 cases of varying pregnancy weeks and perinatal outcomes in the existing literature. Moreover, a better understanding of how infection timing affects maternal–fetal outcomes is of the greatest importance for healthcare professionals to perform risk forecasting and stratification, develop more efficient management strategies, and prevent morbidity and mortality that may develop.

To address these issues, we performed a retrospective study focusing only on COVID-19 positive pregnant women. We aimed to investigate the impact of COVID-19 infection on perinatal outcomes in pregnant women during different phases of pregnancy.

## Methods

### Study design

This study was conducted in seven obstetrics departments affiliated with the Anhui Maternal and Child Health Care Hospital, Hefei, China, between December 7, 2022 and April 30, 2023. Before December 6, 2022, universal COVID-19 screening was implemented for nearly 2 months in our region. On December 7, universal COVID-19 screening was canceled, except for symptomatic patients or individual requests. However, our hospital still requires all pregnant women to undergo COVID-19 screening upon admission. As our hospital is a tertiary-level health-care facility that has played a major role in the management of COVID-19 pregnant women, therefore, we can recruit most of COVID-19-infected women hospitalized during the study period. In December 2020, China started to vaccinate COVID-19 vaccine among high risk populations, and in January 2021, it started to vaccinate the whole population. By December 2021, the vaccination rate of COVID-19 vaccine in Hefei, Anhui Province reached 92.7%. So, all the admitted women have a history of vaccination.

Due to the retrospective design of the study, the need to obtain informed consent from eligible women was waived, and this study was approved by the institutional ethics committee of Anhui Maternal and Child Health Care Hospital.

The inclusion criteria were all women with singleton pregnancies with laboratory-confirmed COVID-19 (as identified by reverse transcriptase-polymerase chain reaction assay of throat swab samples) during the current pregnancy within the study period. Gestational age was confirmed from an ultrasound during the first-trimester of screening.

The exclusion criteria were multiple pregnancies, intrauterine fetal death, pregnancies complicated by internal or surgical complications. Other exclusion criteria were patients who had been diagnosed with preeclampsia or gestational diabetes mellitus (GDM) before their COVID-19 infection, incomplete data and postpartum confirmed COVID-19.

Pregnant women were divided into four groups based on the gestational age of maternal infection: Group 1, infected during 14–27^+6^ weeks gestation (175 cases); Group 2, infected during 28–36^+6^ weeks gestation (176 cases); Group 3, infected during 37–39^+6^ weeks gestation (200 cases); and Group 4, infected with gestational weeks ≥ 40 weeks (60 cases).

Positive women were categorized as asymptomatic, mild, moderate, or severe, according to symptom severity. Mild COVID-19 refers to fever, cough, or change in taste or smell without dyspnea. Moderate or severe infection is defined as the presence of other upper respiratory symptoms or need for advanced oxygen support, high-flow nasal cannula, noninvasive ventilation, or mechanical ventilation, respiratory or organ failure that required admission to the intensive care unit, or the presence of shock.

All COVID-19 cases were managed according to national guidelines for COVID-19 during pregnancy. The timing and mode of delivery in all positive women were determined mainly based on obstetric indications. COVID-19-affected patients were delivered in an isolation ward with strict infection protection measures for patients and attending staff throughout delivery.

Information on demographic and clinical characteristics and perinatal outcomes was extracted from medical files, including maternal age, body mass index (BMI), gravidity, parity, fever, pregnancy complications (preeclampsia, gestational diabetes mellitus, and premature rupture of membranes), gestational age at infection, gestational age at delivery, and mode of delivery. Neonatal outcomes considered were preterm birth, birth weight, Apgar score < 7 at 5 min, neonatal intensive care unit (NICU) admission, and small for gestational age (SGA).

### Definitions

Preterm birth was defined as birth at ≥ 28 weeks but less than 37 weeks of gestation.

Preeclampsia is defined as a blood pressure reading of 140/90 mmHg or greater, measured on two separate times 4 h apart after 20 weeks of gestation, co-existing with proteinuria, or without proteinuria but accompanied by at least one of the following end-organ dysfunctions: renal involvement, impaired liver function, pulmonary edema, and prodromal signs, such as visual and cerebral symptoms [14].

Gestational diabetes mellitus (GDM) refers to abnormal glucose intolerance of varying degrees, which is first diagnosed in the second or third trimester of pregnancy [15]. The diagnostic criteria of GDM used in China are a 2 h 75 g oral glucose tolerance test (OGTT) performed during the 24th–28th gestational weeks in all pregnant women without overt diabetes. The GDM could be diagnosed if one or more of the OGTT plasma glucose values meets or exceeds the following cutoff values: 5.1 mmol/L at fasting; 10.0 mmol/L at 1 h; and 8.5 mmol/L at 2 h.

Premature rupture of membranes (PROM) is defined as the rupture of membranes before the onset of labor [16].

Small for gestational age (SGA) refers to a fetal birth weight less than the 10th percentile for gestational age, depending on the local population growth charts [17].

### Statistical analysis

Statistical analyses were performed using SPSS (version 13.0; SPSS Inc, Chicago, IL, USA). The counting variables were expressed as percentages. Continuous variables were expressed as mean and standard deviation (SD). The four groups were compared using the F-test for continuous variables and the chi-square test for categorical variables, as appropriate. Multivariate logistic regression analysis was conducted to identify the association between gestational age at COVID-19 onset and various variables. Odds ratios (OR) and 95% confidence intervals (CI) were used to quantify the association. Statistical significance was set at *P* < 0.05.

## Results

During the study period, 611 patients met the study inclusion criteria. Among the pregnant women, 161 were asymptomatic and 450 were symptomatic. All symptomatic women were classified as mild, including fever, cough, or change in taste or smell without dyspnea, and none were classified as having moderate or severe symptoms. The most common presenting symptom was fever (n = 435, 71.2%).

A comparison of patient characteristics between the four groups is presented in Table 1. No clinically significant differences were observed among the four groups in terms of maternal age, BMI, gravidity and multiparity. (*P* > 0.05).

**Table 1:** Comparison of demographic characteristics of the study population between four groups

Variables	Group1 (N = 175)	Group2 (N = 176)	Group3 (N = 200)	Group 4 (N = 60)	*F/χ2*	*P*
Maternal age (years)	30 ± 3.9	30.8 ± 4.3	30.3 ± 3.7	30.1 ± 3.8	1.22	0.300
BMI (kg/m^2^)	21.8 ± 3	21.8 ± 3.7	21.6 ± 3.1	21.6 ± 2.7	0.25	0.862
Gravidity (n, %)					0.67	0.716
1	84 (48)	61 (34.7)	92 (46)	30 (50)		
2	51 (29.1)	59 (33.5)	60(30)	20 (33.3)		
≥ 3	40 (22.9)	56 (31.8)	48 (24)	10 (16.7)		
Multiparity (n, %)	62 (35.4)	81 (46.0)	69 (34.5)	18 (30.0)	0.98	0.322

BMI: body mass index; data are expressed as mean ± SD or n (%); *P* < 0.05, considered statistically significant. Group 1 participants were infected with COVID-19 during 14–27^+6^ weeks gestation; Group 2 participants were infected with COVID-19 during 28–36^+6^ weeks gestation; Group 3 participants were infected with COVID-19 during 37–39^+6^ weeks gestation; Group 4 participants COVID-19 infection with gestational weeks ≥ 40 weeks

A comparison of the clinical course and birth outcomes among the four groups is shown in Table 2. There were no statistically significant differences in the rates of PROM and vaginal delivery between the groups (*P* > 0.05). Significant differences were observed in the mean gestational age at infection and mean gestational age at delivery among the four groups (*P* < 0.05). Pregnant women who tested positive for COVID-19 in groups 1 and 2 were significantly more likely to have fever than those who tested positive in groups 3 and 4 (*P* < 0.05). Moreover, as gestational age increased, the proportion of preeclampsia and GDM decreased (*P* < 0.05).

**Table 2:** Comparison of pregnancy characteristics and delivery outcomes of the study population between four groups

Variables	Group1 (N = 175)	Group2 (N = 176)	Group3 (N = 200)	Group 4 (N = 60)	*F/χ2*	*P*
Gestational age at infection (weeks)	24.2 ± 2	33.4 ± 2.4	38.5 ± 0.8	40.3 ± 0.3	2519.91	< 0.001
Gestational age at delivery (weeks)	39.2 ± 1.2	38.6 ± 1.8	39.1 ± 0.8	40.5 ± 0.4	33.48	< 0.001
Fever (n, %)	150 (85.7)	158 (89.8)	111 (55.5)	16 (26.7)	101.89	< 0.001
GDM (n, %)	39 (22.3)	32 (18.2)	29 (14.5)	5 (8.3)	7.50	0.006
Pre-eclampsia (n, %)	28 (16)	23 (13.1)	15 (7.5)	4 (6.7)	7.92	0.005
PROM (n, %)	31 (17.7)	40 (22.7)	42 (21.0)	6 (10.0)	0.30	0.583
Vaginal delivery (n, %)	117 (66.9)	155 (88.1)	140 (70)	47 (78.3)	0.60	0.439

GDM: gestational diabetes mellitus; PROM: Premature rupture of membranes; data are expressed as mean ± SD or n (%); *P* < 0.05, considered statistically significant. Group 1 participants were infected with COVID-19 during 14–27^+6^ weeks gestation; Group 2 participants were infected with COVID-19 during 28–36^+6^ weeks gestation; Group 3 participants were infected with COVID-19 during 37–39^+6^ weeks gestation; Group 4 participants COVID-19 infection with gestational weeks ≥ 40 weeks

The comparison of neonatal outcomes among the four groups is presented in Table 3. There were no differences among the four groups in the incidence of SGA and Apgar score < 7 at 5 min (*P* > 0.05). A significantly higher frequency of NICU admission was observed in group 2, followed by group1, 4 and 3. Furthermore, 7 and 22 premature births occurred in group 1 and group 2, respectively, and no preterm births were found in groups 3 and 4. There were 2 and 4 cases of iatrogenic prematurity in group 1 and group 2, respectively. The preterm delivery rate was significantly higher in the newborns in group 2 than in those in group 1 (*P* < 0.05). Additionally, there was a significant inter-group difference in terms of the mean birth weight (*P* < 0.05), although they were all within the normal range.

**Table 3:** Comparison of neonatal outcomes between four groups

Variables	Group1 (N = 175)	Group2 (N = 176)	Group3 (N = 200)	Group 4 (N = 60)	*F/χ2*	*P*
Birth weight (g)	3292.6 ± 439.7	3274.1 ± 452.5	3365.1 ± 406.2	3544.8 ± 330.4	7.04	< 0.001
Premature birth (n, %)	7 (4)	22 (12.5)			7.33	0.007
Asphyxia (n, %)	2 (1.1)	1 (0.6)	1 (0.5)		1.00	0.316
NICU admission (n, %)	14 (8)	18 (10.2)	3 (1.5)	1 (1.7)	9.54	0.002
SGA (n, %)	14 (8.0)	10 (5.7)	18 (9.0)	3 (5.0)	0.01	0.914

NICU: neonatal intensive care unit; SGA: small for gestational age; data are expressed as mean ± SD or n (%); *P* < 0.05, considered statistically significant. Group 1 participants were infected with COVID-19 during 14–27^+6^ weeks gestation; Group 2 participants were infected with COVID-19 during 28–36^+6^ weeks gestation; Group 3 participants were infected with COVID-19 during 37–39^+6^ weeks gestation; Group 4 participants COVID-19 infection with gestational weeks ≥ 40 weeks

Table 4 summarizes the results of the multivariate logistic regression analysis of the association between gestational age at infection and various parameters. After adjusting for variables including maternal age, BMI, gravidity, parity, gestational age at delivery, and fever. A negative statistically significant correlation was found between gestational age at diagnosis and the risk of preeclampsia and GDM (OR = 0.955; 95% CI 0.916–0.996 and OR = 0.960; 95% CI 0.927–0.995, respectively, *P* < 0.05). However, no significant correlation was present between the gestational age at infection and the remaining parameters (*P* > 0.05).

**Table 4:** Multivariate logistic regression analysis of the association between gestational age at COVID-19 infection and perinatal outcomes

Variables	β	SE	Wald*χ*^2^	*P*	OR	95% CI
GDM (n, %)	− 0.041	0.018	5.056	0.025	0.960	0.927–0.995
Pre-eclampsia (n, %)	− 0.046	0.022	4.549	0.033	0.955	0.916–0.996
PROM (n, %)	0.009	0.018	0.271	0.603	1.009	0.975–1.045
Vaginal delivery (n, %)	0.018	0.016	1.302	0.254	1.018	0.987–1.050
Premature birth (n, %)	− 0.009	0.084	0.012	0.913	0.991	0.841–1.168
Asphyxia (n, %)	− 0.071	0.093	0.573	0.449	0.932	0.776–1.119
NICU admission (n, %)	− 0.058	0.032	3.223	0.073	0.994	0.885–1.005
SGA (n, %)	− 0.005	0.026	0.030	0.863	0.995	0.946–1.048

GDM: gestational diabetes mellitus; PROM: Premature rupture of membranes; NICU: neonatal intensive care unit; SGA: small for gestational age; SE: standard error; OR: odds ratio; CI: confidence interval; *P* < 0.05, were considered statistically significant

## Discussion

A novel finding of the present study is that gestational weeks of maternal COVID-19 infection are negatively correlated with the occurrence of GDM and preeclampsia. Another interesting finding is that composite adverse fetal outcomes are not associated with the pregnancy stages of maternal infection.

To date, the relationship between maternal COVID-19 infection and preeclampsia is a popular topic that has been the focus of numerous studies. From a clinical point of view, pregnant women with COVID-19 infection are at an increased risk of preeclampsia. The underlying mechanisms seem multifactorial, and we believe that there are two possible explanations. One possible explanation is the well-known angiotensin-converting enzyme 2 (ACE2), which plays a crucial role as a receptor for SARS-CoV-2 to enter the host cells [18]. ACE2 is widely present in pulmonary epithelial cells, endothelial cells, and other cell types [19]. Endothelial infection can promote endothelial injury, multi-organ inflammation and microthrombus formation in different vascular zones [20–22]. In contrast, endothelial cell dysfunction and intravascular inflammation have emerged as the main pathophysiological features of the development of preeclampsia [23]. Another explanation is that ACE2 plays a pivotal role in the renin-angiotensin system (RAS) in the regulation of maternal hemodynamic adaptations [24]. When SARS-CoV-2 binds to the ACE2 receptor, its expression is reduced. Consequently, RAS is activated, which in turn could cause adverse hemodynamics in pregnant women [25]. Consequently, these alterations might contribute to the pathogenesis of preeclampsia [26, 27]. Taken together, COVID-19 infection and preeclampsia share several common pathways, and there are sufficient data correlating antenatal COVID-19 infection with an increased risk of preeclampsia [6, 28, 29]. In contrast to their research, we aim to fill research gaps in the existing literature by evaluating the length of in-utero exposure and the risk of preeclampsia. The findings of the study demonstrate that the smaller the gestational age of infection, the greater the probability of developing preeclampsia, for a 4.5% decrease per additional week, which is supported by the fact that ACE2 expression is related to immaturity in the placenta, while the expression of these proteins is low in term placentas [30].

With regard to GDM, most of the studies available on COVID-19 have evaluated the impact of infection on GDM, with an enormous amount of published data demonstrating an increased risk of developing GDM [31–33]. There are clear explanations for this observation: (1) Feelings of loneliness and worry are intensified during the COVID-19 epidemic lockdown period [34], which are among the most common factors that worsen hyperglycemia [35]. (2) Various lockdowns are associated with a reduction in physical activity [36] and substantially impacted dietary habits [37], regular exercise and a healthy diet during pregnancy are known to reduce the risk of GDM [38, 39]. (3) Mechanisms of SARS-CoV-2 infection are characterized by cytokine storm syndrome, including elevated levels of interleukin-6 [40], and interleukin-6 plays a role in the development of insulin resistance and hyperglycemia [41]. (4) The ACE2 receptor is expressed on the surface of pancreatic beta cells and SARS-CoV can mediate pancreatic islet cell damage by binding to the ACE2 receptor, which in turn leads to GDM. To date, very little evidence has been published regarding the impact of COVID-19 on the incidence of GDM stratified by gestational age of infection. In a retrospective analysis of prospectively collected data on uninfected and asymptomatic women, the authors concluded that pandemic-related control measures may have affected exercise levels in pregnant women, especially in those in their first and early second trimesters, thus ultimately favoring GDM [42]. This finding was further supported by a retrospective cross-sectional study, indicating that SARS-CoV-2 infection at the time of hospitalization for delivery did not significantly impact GDM because the parameter is a function of gestational age and changes over a longer period [43]. In our study, we demonstrated that early COVID-19 infection is associated with a higher risk of developing GDM in pregnant women, and an increase of 1 week in gestational age decreased the risk of GDM by 4.0%. However, the study design differed markedly from that reported in the literature. Our data were collected during a unique period in which the policies of lockdown and universal COVID-19 screening have just been canceled. Notably, pregnant women experience unprecedented anxiety and worry levels due to the risk of infection and the possibility of vertical transmission of the disease in such circumstances. Additionally, the categorization was based on the week of gestation in which COVID-19 infection was confirmed. Nonetheless, findings from this study provide valuable insights into the experience of COVID-19 infection, which reinforces the importance of gestational age at COVID-19 infection in determining the outcome of pregnancy.

Regarding the occurrence of composite adverse fetal outcomes, the results of the study demonstrate that SARS-CoV-2 infection during early stages of pregnancy has increased rates of preterm birth and NICU admission, and there is no difference in the rate of SGA, while these differences are not associated with the gestational age of maternal infection. Perinatal outcomes are certainly one of the main concerns of maternal–fetal specialists. To date, a massive research effort to better understand the impact of COVID-19 infection on perinatal outcomes has resulted in an extraordinary volume of literature published, with increasing reports advising on the adverse effects of COVID-19 infection on premature [44, 45], especially when pregnant women were admitted to the intensive care unit with severe-critical COVID-19, the preterm delivery rate was significantly higher in the non-survivor group [46]. However, our knowledge is limited regarding the influence of the timing of infection on premature birth; only one retrospective cohort study found that first and second trimester maternal SARS-CoV-2 infection was a risk factor for preterm birth [47]. However, our findings failed to demonstrate this association. The discrepancies between the previous study and our study might be explained by the structure of the study group and heterogeneity of the studied populations. The question of why COVID-19 exposure during early pregnancy increases the prevalence of preterm births is not exactly known, but in our study, we hypothesized that the increased incidence of fever in the same time frame may play a pivotal role, as available evidence demonstrated the maternal inflammatory response was significantly associated with a seven-fold increased risk of preterm delivery among the COVID-19-infected group [48]. In addition, interpretation of the higher rates of NICU admission in women with COVID-19 infection earlier in pregnancy requires consideration of the higher incidence of preterm deliveries during this time period, which is supported by the fact that COVID-19 may pose a larger threat to infants through preterm birth [49, 50]. Interestingly, there were no differences in the frequency of SGA among the four groups, and this finding is in agreement with those of other studies that reported no difference in SGA rates regardless of the trimester of infection [51, 52].

This study has several limitations. First, the results of the present study may be limited by its retrospective nature, which may lead to potential sources of bias and misleading results; second, at the time of our study, information on the effects of the virus in the first trimester were sparse, thus the clinical progression of the disease in the first trimester and the effect of the disease on perinatal outcomes could not be determined. However, this study also has a number of strengths. The COVID-19 screening strategy has definite strengths. This permit the capture of undiagnosed asymptomatic infection and mild cases, and the precise timing of COVID-19 infection is ascertained, which could strengthen the magnitude of the associations assessed. Furthermore, data from only one hospital is another robust strength, as it lowers the risk of differences in data processing and hospital practices, such as obstetricians’ experience and guidelines regarding management and follow-up to the women included, and the equipment and technique used for the polymerase Chain Reaction.

In conclusion, from the first cases of COVID-19 infection in late 2019, lack of knowledge about COVID-19 infection in pregnancy has raised urgent questions about the risk of maternal, fetal and neonatal morbidity and mortality. There is an urgent need to establish evidence-based guidance to guide clinical decision-making. Our results underline that early COVID-19 infections carries a substantial risk of adverse perinatal outcomes. All of these findings together underscore and support the importance of early establishment of reinforced obstetric surveillance, routine follow up and close monitoring for the development of GDM and preeclampsia for pregnant women with infection at an early gestational age with hope for better perinatal outcomes.

## Data Availability

The datasets used during the current study are available from 10.6084/m9.figshare.22989677.

## Abbreviations

COVID-19: Coronavirus disease 2019
SARS-CoV-2: Severe acute respiratory syndrome-coronavirus-2
GDM: Gestational diabetes mellitus
BMI: Body mass index
NICU: Neonatal intensive care unit
SGA: Small for gestational age
PROM: Premature rupture of membranes
SD: Standard deviation
OR: Odds ratios
OGTT: Oral glucose tolerance test
CI: Confidence intervals
SE: Standard error
ACE2: Angiotensin-converting enzyme 2
RAS: Renin-angiotensin system
